# Morphea En Coup De Sabre Treated With Combination Hyaluronic Acid Filler and Neuromodulator: A Case Report

**DOI:** 10.7759/cureus.105502

**Published:** 2026-03-19

**Authors:** Colton Jensen, B Kent Remington

**Affiliations:** 1 Dermatology, University of Alberta, Edmonton, CAN; 2 Dermatology, Remington Laser Dermatology Centre, Calgary, CAN

**Keywords:** botulinum toxin, en coup de sabre morphea, hyaluronic acid filler, linear scleroderma, microcannula, neuromodulator, prabotulinumtoxin-a

## Abstract

Morphea en coup de sabre is a localized scleroderma variant that often leaves permanent, disfiguring frontoparietal atrophy after disease stabilization. We report a 27-year-old woman with four stable, linear, atrophic forehead plaques consistent with burned-out morphea en coup de sabre who sought cosmetic improvement. Targeted prabotulinumtoxin-A was administered to hyperfunctional temporalis, corrugator supercilii, and depressor supercilii muscles to reduce dynamic deforming forces. Two weeks later, hyaluronic acid filler was delivered via a blunt-tipped microcannula on the periosteal plane in a zonal pattern to restore contour. This combination approach provided immediate, natural-appearing correction maintained with small-volume retreatments every six to 12 months without complications. This case illustrates that neuromodulator and hyaluronic acid filler, delivered with a cannula, may offer a safe, minimally invasive option for aesthetic rehabilitation of burned-out morphea en coup de sabre by addressing muscle imbalance while reshaping and contouring the forehead.

## Introduction

Morphea en coup de sabre (also known as frontoparietal linear morphea, linear morphea en coup de sabre, or en coup de sabre) is a variant of localized scleroderma presenting as a linear, atrophic depression on the frontoparietal region of the forehead. It evolves from a unilateral, inflammatory, linear, indurated plaque to an ivory, indurated scar with a violaceous margin [1]. The condition occasionally involves underlying muscle, bone, and rarely the meninges and brain [2,3]. In some cases, the disease may manifest with neurologic or ophthalmologic symptoms [4,5]. The etiology of morphea en coup de sabre is formally unknown, though disease progression is likely regulated by a combination of autoimmune and genetic components [6]. Burned-out morphea en coup de sabre leaves permanent atrophic scars, causing facial asymmetry and cosmetic distress. Though self-limited (active phase two to five years), residual cosmetic disfigurement profoundly impacts quality of life, driving demand for reconstructive options.

Treatments for burned-out lesions have not been standardized, and there is a broad spectrum of results from treatment with surgical excision, autologous fat grafting, autologous bone grafting, placement of synthetic material, micronized acellular dermal matrix, and platelet-rich plasma [7-9]. We report a case of stable morphea en coup de sabre successfully treated with a combination of neuromodulator and hyaluronic acid injections via a blunt-tipped microcannula. 

## Case presentation

**Figure 1:**
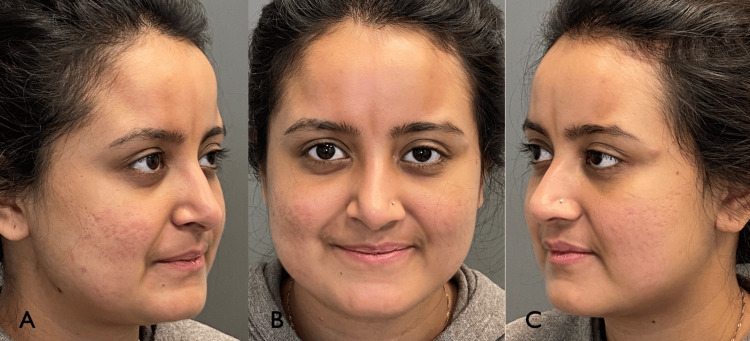
Patient three-quarter views (A,C) and frontal (B) and prior to treatment showing four linear atrophic plaques on the forehead.

**Figure 2:**
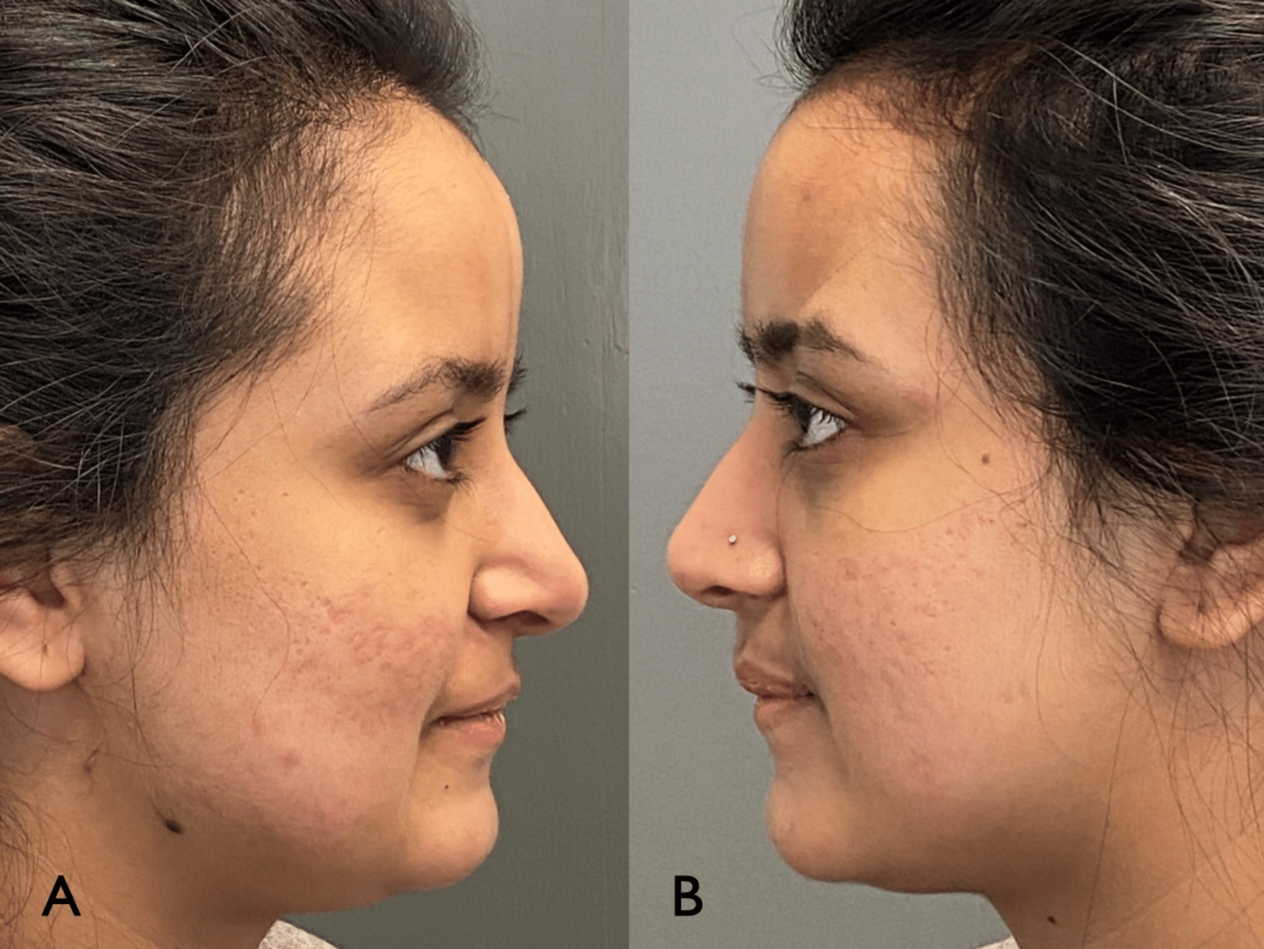
Patient right (A) and left (B) profile prior to treatment.

A 27-year-old female presented with an approximately 14-year history of linear, atrophic plaques in four separate zones on her forehead in keeping with burned-out morphea en coup de sabre. The areas were stable and asymptomatic, and the patient was seeking an improved cosmetic appearance for these disfiguring lesions. Of note, her hypertrophied temporalis muscles and hyperactive corrugator supercilii and depressor supercilii muscles accentuated the depth of her morphea en coup de sabre plaques (Figures 1, 2). 

**Figure 3:**
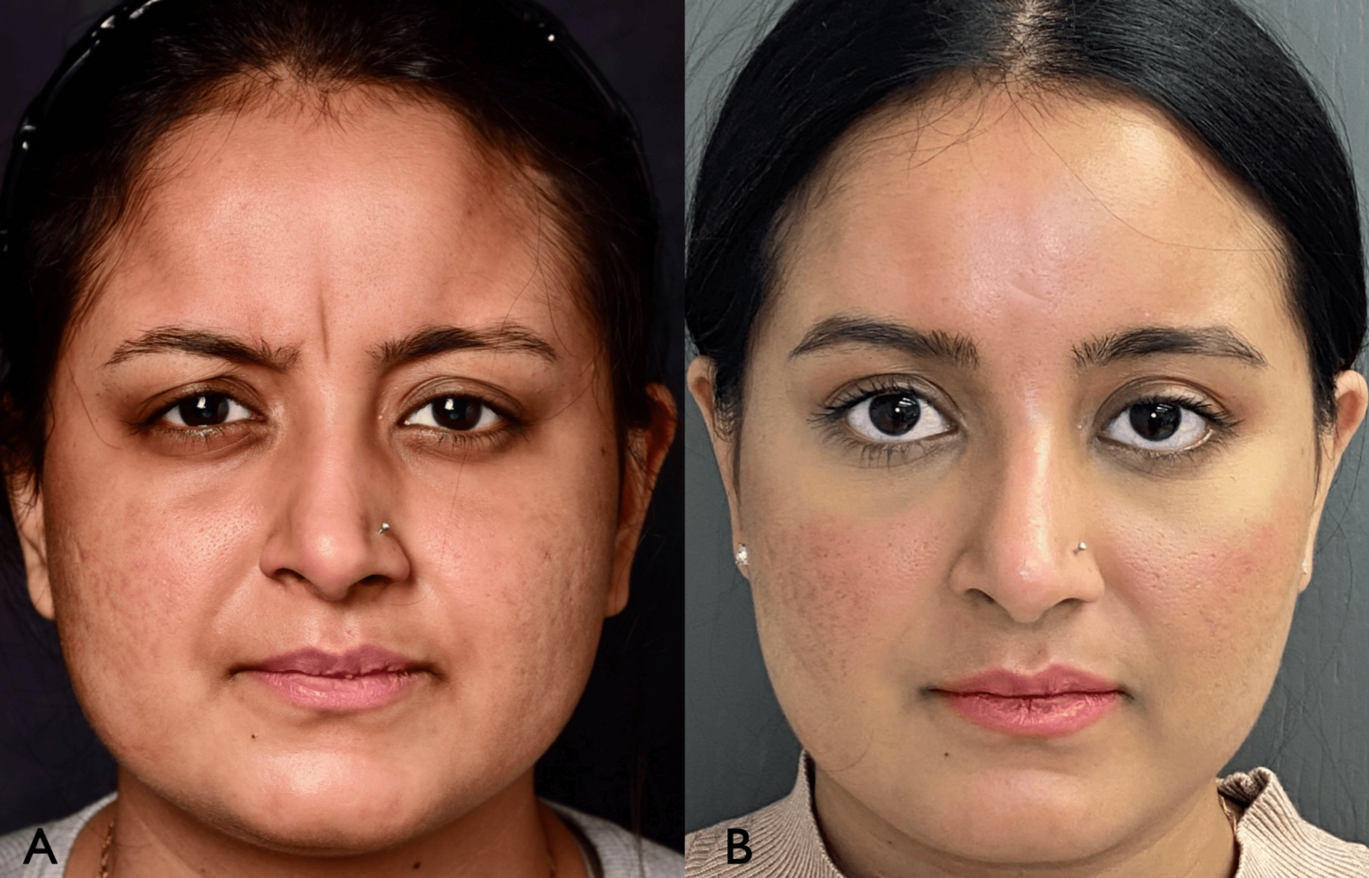
Patient prior to treatment (A). Repeat photograph 4 weeks after first neuromodulator injections and 2 weeks after first hyaluronic acid filler injections (B).

**Figure 4:**
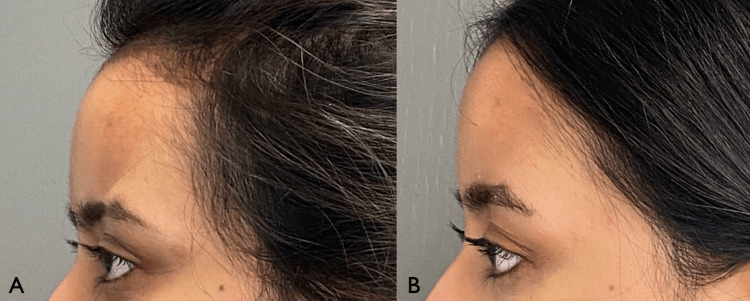
Patient left profile prior to treatment (A). Repeat photograph 4 weeks after first neuromodulator injections and 2 weeks after first hyaluronic acid filler injections (B).

**Figure 5:**
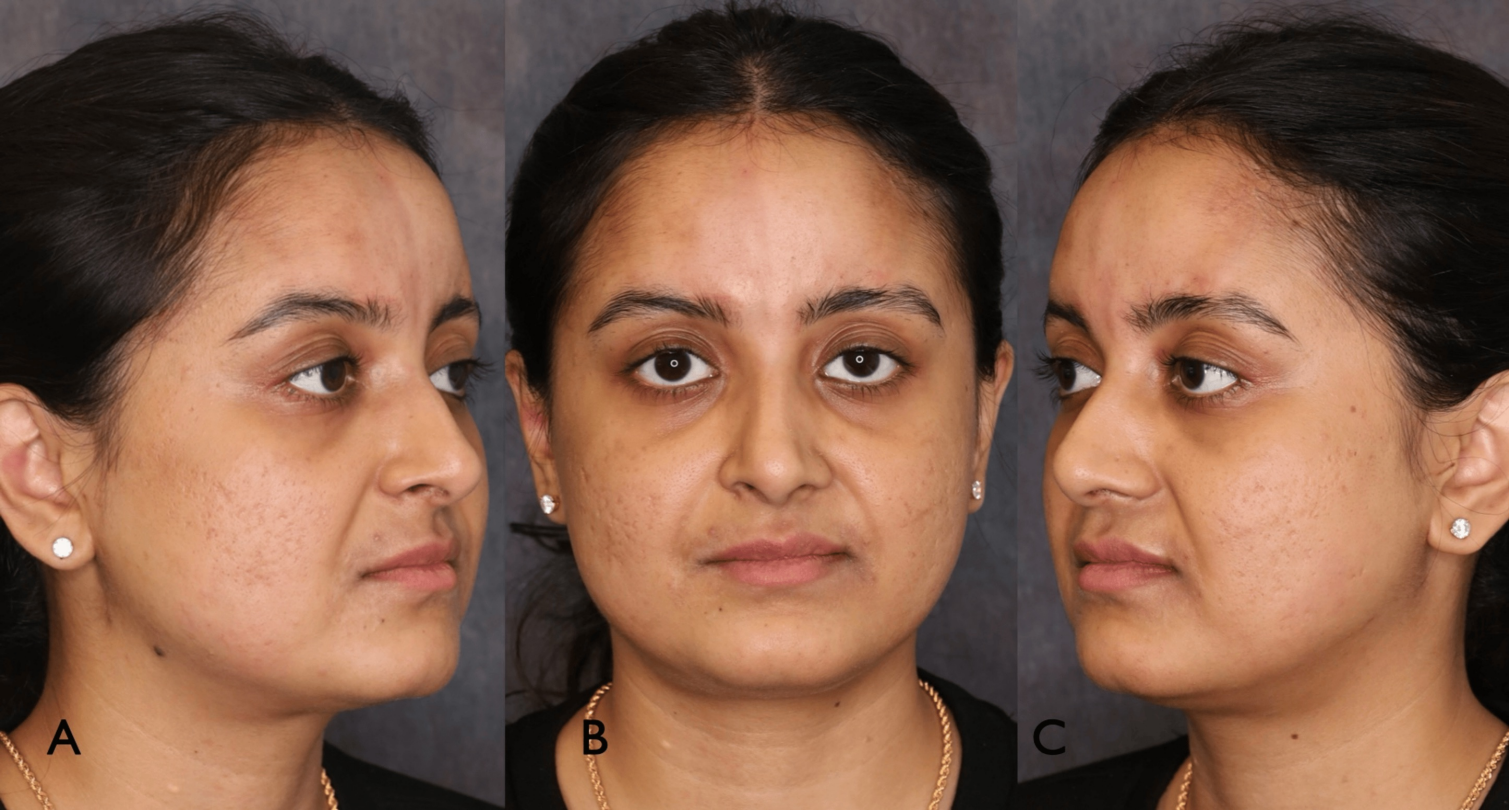
Patient three-quarter views (A,C) and frontal (B) and approximately 5 years after first treatment.

**Figure 6:**
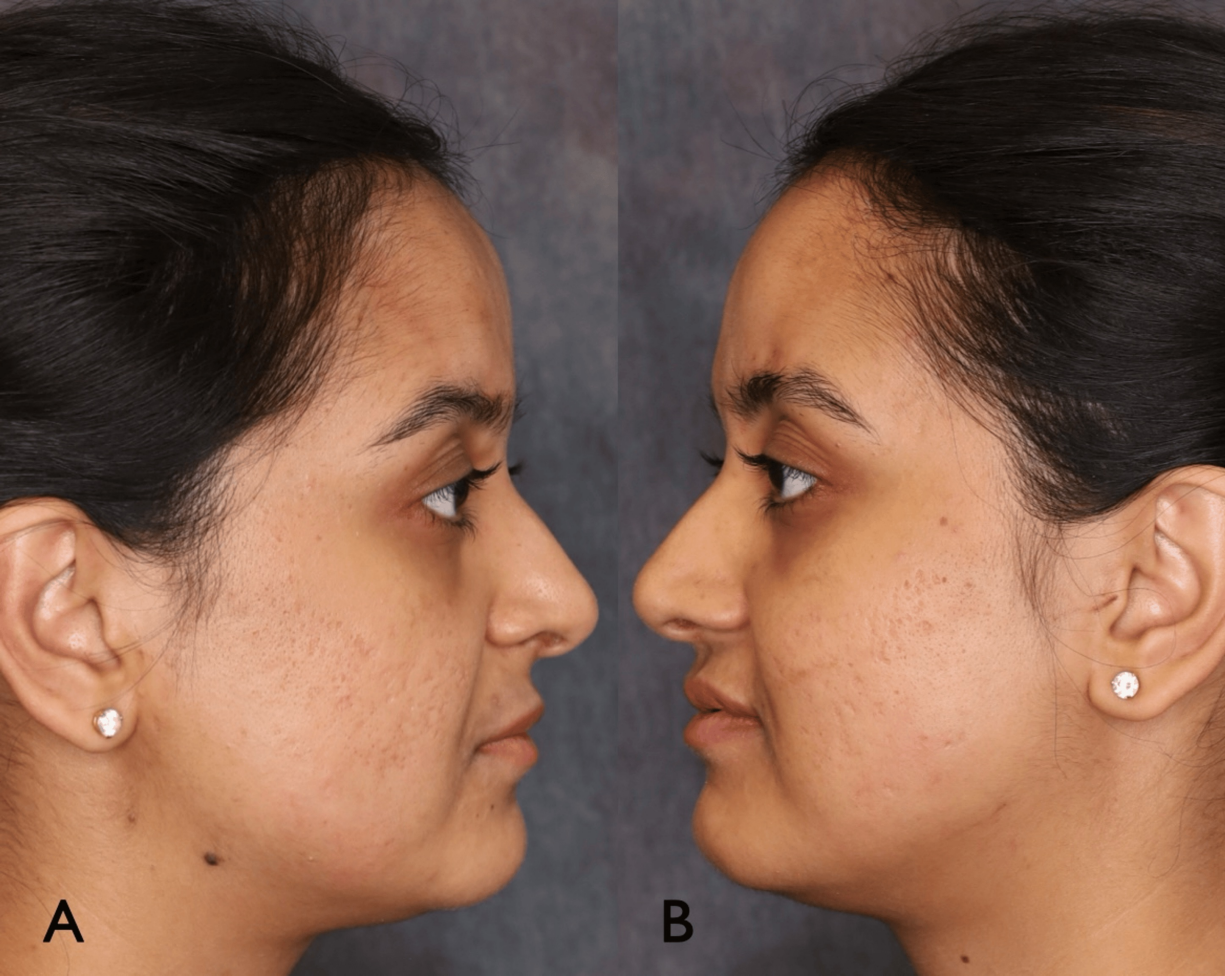
Patient right (A) and left (B) profile approximately 5 years after first treatment.

Following detailed photography, treatment was initiated. First, prabotulinumtoxin-A (Nuceiva; Evolus, Newport Beach, CA) was reconstituted to 5 U per 0.1 mL and delivered using a dose saver 1 mL syringe with a 32-gauge 1/2 needle was used to modify the bilateral temporalis, corrugator supercilii, and depressor supercilii muscles. Two weeks later, the patient was seen in follow-up. At that time, a 23-gauge trocar was inserted near the frontal hairline, and a 25-gauge 50 mm cannula was used to break up the fibrotic tissue and create a space via blunt dissection both under and beside the defects. The same cannula was then used to inject 1.5 mL of TEOSYAL® PureSense Redensity 2 (Teoxane, Geneva, Switzerland) blended with normal saline into each of the four zones; the product was injected both anterograde and retrograde onto the periosteal plane in a zone pattern instead of the more traditional linear band pattern. The injected site was then massaged with cool ultrasound gel. Cosmetic improvements were immediate and maintained with repeat small-volume injections every six to 12 months (Figures 3-6).

## Discussion

Morphea en coup de sabre, a subtype of linear scleroderma, typically leaves permanent, disfiguring, atrophic scars on the forehead. Multiple treatment modalities have been proposed to treat the active phase of the disease, including methotrexate, systemic corticosteroids, cyclosporine, mycophenolate mofetil, hydroxychloroquine, abatacept, tocilizumab, interferon gamma, ultraviolet radiation, and pulsed dye laser [7]. Additionally, a plethora of treatments for the residual atrophic scars have been proposed, including surgical excision, fat grafting, and injectable products, including micronized acellular dermal matrix, polymethylmethacrylate, poly-L-lactic acid, calcium hydroxyapatite, platelet-rich plasma, and hyaluronic acid [7-9]. 

Ideally, treatment should employ a modality that is effective, tolerable, accessible, and safe. Soft tissue fillers provide a less invasive option accessible to most patients; specifically, hyaluronic acid fillers confer several advantages. In addition to adding supplemental glycosaminoglycan with the injection itself, hyaluronic acid filler stimulates the endogenous production of collagen, elastin, and ground substance in the treated areas. With proper technique, it can be carefully placed to provide not only immediate correction of the targeted atrophy, but also provide long-lasting results. Despite the paucity of reports using hyaluronic acid filler for the treatment of morphea en coup de sabre, we found one report of administering hyaluronic acid filler using a blunt-tipped cannula [10]. The use of a cannula as an alternative to sharp needle injection is preferred for morphea en coup de sabre, given the higher risk of intravascular compromise on the forehead; furthermore, it is less traumatic with a reduced risk of pain, swelling, and bruising. Though more permanent therapeutic options may be enticing, the unpredictability of other treatment options in combination with the possible complications in this zone confers unnecessary risk; hyaluronic acid filler injections for morphea en coup de sabre may be a safer choice. As highlighted by Choski et al., additional benefits of choosing hyaluronic acid filler include ease of injection, the availability and diversity of filler materials, and minimal social downtime [11].

Patients with burned-out morphea en coup de sabre may exhibit altered activity of the facial expression muscles due to the tethering effect of the atrophic plaques. This can result in asymmetric muscle activation, further accentuating facial asymmetry and dystrophy. In such cases, combination therapy with hyaluronic acid filler and a neuromodulator may be considered to achieve a complementary neuromyomodulatory effect within the affected area.

Ultimately, a combination of hyaluronic acid filler and neuromodulator injection may be a preferred treatment option for burned-out morphea en coup de sabre. Based on the results obtained in the above patient, combination treatment with hyaluronic acid filler and neuromodulator is an effective alternative to more invasive procedures. 

## Conclusions

This case demonstrates that targeted neuromodulator injections to hyperfunctional facial muscles, combined with hyaluronic acid filler delivered via blunt-tipped microcannula on the periosteal plane, offer a safe, minimally invasive, and effective strategy for aesthetic rehabilitation of burned-out morphea en coup de sabre. By addressing both dynamic deforming forces and static atrophic contour deficits, this approach achieved immediate, natural-appearing correction with sustained results through small-volume maintenance treatments every six to 12 months. Furthermore, the microcannula and technique of creating a space before retrograde and anterograde injections limit the risk of complications. These findings support combination neuromodulation and hyaluronic acid filler injection as a promising first-line option for managing residual facial asymmetry in stable morphea en coup de sabre.
